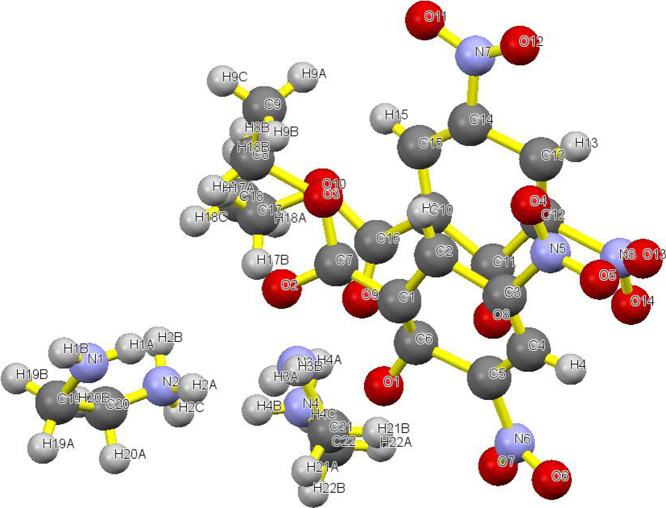# 2-Aminoethanaminium 2-(ethoxycarbonyl)-4,6-dinitrophenolate as a greener route in reducing sugar quantification

**DOI:** 10.1016/j.mex.2018.05.017

**Published:** 2018-06-05

**Authors:** Abdel-Naser A. Zohri, Mohamed Abdelazim, Sara Ibrahim

**Affiliations:** a Faculty of Science, Botany and Microbiology Department, Assiut University, Assiut, 71516, Egypt; b Chemical and Biotechnological Laboratories, Assiut University, Sugar Industry Technology Research Institute, Assiut, 71516, Egypt

**Keywords:** AED in reducing sugar quantification, Organic proton transfer salts, AED, DNS, Application in reducing sugar determination, Greener route

## Abstract

3,5-dinitrosalicylic acid (DNS) reducing sugar assay is the most convenient method for quantification of total reducing sugar in biomass hydrolysate, fermentation samples, sugar industry and biotechnology laboratories. The dimeric proton transfer salt 2-aminoethanaminium 2-(ethoxycarbonyl)-4,6-dinitrophenolate (AED) is an intensely colored derivative of DNS and in turn its reduced form intense color showed a superior properties in reducing sugar quantification eliminating phenol and rochelle salt additives using the same practical methodology of DNS giving an overall methodology advantageous than using DNS assay as a greener route.

The proton transfer salt has already been X-ray imaged and deposited in Cambridge Crystallographic Data Centre CCDC 1441586 and a comparison was done between DNS and this salt using a salt and sodium hydroxide concentrations as the same as DNS assay as well as the latter phenol-rochelle salt free environment giving correlation coefficient 0.999 and absorptivity nearly two thirds the obtained in case of use DNS assay with added phenol for enhancing absorptivity and rochelle salt for produced color stabilization for the same detection range 0.1-0.5 mg/ml according to Miller procedures. The sensitivity and the reduced form color stability of this proton transfer salt could be interpreted on both of its molecular structure has a double oxidizing groups as well as it has an intense color as compared with DNS and a good reduced form solubility respectively.

• DNS in reducing sugar quantification, Miller procedures involving phenol and rochelle salt addition.
• AED in reducing sugar quantification as the same detection range as DNS with the elimination of phenol and rochelle salt from the assay.
• A greener convenient route in reducing sugar quantification.

## Graphical abstract

**Figure:**
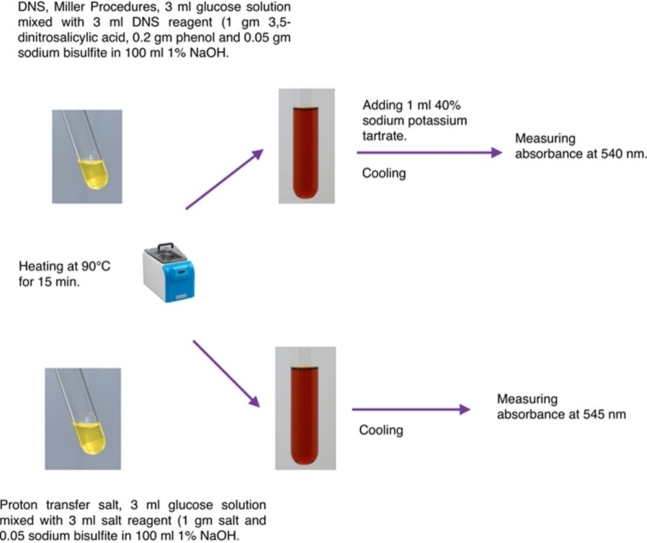

**Specification Table**

**Table:** 

Subject area	Chemistry
More specific subject area	*Spectrophotometric determination of reducing sugars.*
Method name	*AED in reducing sugar quantification.*
Name and reference of original method	*Use of Dinitrosalicylic Acid Reagent for Determination of Reducing Sugar.* *G. L. Miller, Anal. Chem, 1959, 31(3), 426-428*

## Method details

DNS, Original Miller Method.

Reagents and Equipments:

- 3,5-dinitrosalicylic acid.
- Sodium hydroxide.
- Phenol.
- Rochelle salt, sodium potassium tartrate.
- Sodium bisulfite.
- Water bath.
- Spectrophotometer.

Procedures:

3 ml of glucose solution 0.1-0.5 mg/ml is mixed with 3 ml of DNS reagent (1 gm of DNS dissolved in 100 ml 1% sodium hydroxide contains 0.2 gm phenol and 0.05 gm sodium bisulfite), the reaction mixture was heated at 90 °C for 15 min and 1 ml of 40% sodium potassium tartrate is added then cooled and the absorbance is measured at 540 nm.

AED method.

Reagents and Equibments:

- AED is prepared by the reaction of ethyl 3,5-dinitrosalicylate [1] using ethyl salicylate instead of methyl salicylate with ethylenediamine in hot ethanol for 5 min then collecting the yellow precipitate and recrystalized from water.
- Sodium hydroxide.
- Sodium bisulfite.
- Water bath.
- Spectrophotometer.

Procedures:

3 ml of glucose solution 0.1-0.5 mg/ml is mixed with 3 ml of AED reagent (1 gm AED dissolved in 100 ml 1% sodium hydroxide contains 0.05 gm sodium bisulfite), the reaction mixure was heated at 90 °C for 15 min then cooled and the absorbance is measured at 545 nm.

In this laboratory [2] the reducing sugar is extensively and routinely analyzed in sugarcane molasses, biomass pretreatment hydrolysate, and woody residual enzymatic hydrolysate and due to the lower Pk_a_ of DNS that might be in relation to skin irritation in case of contact and its relatively high price we investigated this reagent AED under study in comparison with DNS in reducing sugar quantification prepared simply starting with a cheap available chemicals, ethyl salicylate, sulfuric acid, nitric acid and ethylenediamine and its intense non reduced and reduced color was successfully utilized to substitute DNS as well as the elimination of phenol a skin burning compound and rochelle salt from the redox reaction mixture as shown in the following table.

Comparison of DNS and its ethyl ester ethylendiamine proton transfer salt, AED, in assay parameters.

**Table:** 

	AED	DNS
Testing sugar range mg/ml	0.1-0.5	0.1-0.5
Reagent concentration	1 gm in 100 ml 1% NaOH	1 gm in 100 ml 1% NaOH
Phenol addtion	not added	Must be added, 0.2 gm.
Sodium bisulfite	Added, 0.05 gm.	Added, 0.05 gm.
Rochelle salt	Not added	Must be added, 1 ml of 40% at the end of heating.
Milliliters of reagent used	3 ml.	3 ml.
Milliliters of glucose used	3 ml.	3 ml.
Temperature C	90	90
Time	15 min.	15 min.
Correlation coeffecient	0.999	0.999
Slope	3.36	4.75
Standard Glucose Solution 0.1%	0.106%	0.104%

Additional information:

The presence of four nitro groups in the molecular structure of 2-aminoethanaminium 2-(ethoxycarbonyl)-4,6-dinitrophenolate [3] is responsible for its reduction under alkaline conditions at 90 °C by the reducing action of glucose for 15 min in a basic medium giving a red optical density directly proportional to glucose concentration at 545 nm. Making a comparison between this salt and DNS, Miller procedures [4], in reducing sugar quantification concluded the higher sensitivity indicated by the absence of phenol in the redox reaction to obtain a red reduced color as well as the stability of this produced red optical density upto 6 h with a slight decrease in absorbance value noticed after 2 h then relatively became constant without any addition of both of phenol responsible for raising absorbance value fifth times in glucose concentration range 0.1-0.5 mg/ml than its absence and Rochelle salt responsible for reduced form color stabilization as it was reported in DNS Miller methodology reveals a superior greener route in reducing sugar analysis using this organic proton transfer salt.

**Fig. 1:**
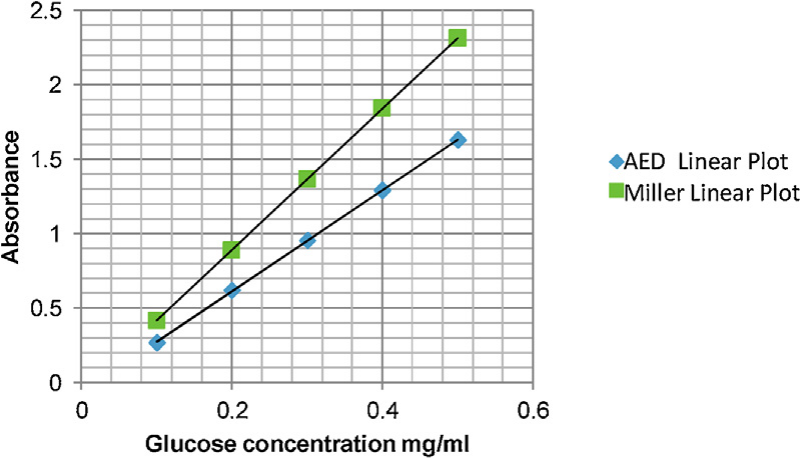
Beer-Lamber plot for 3,5-dinitrosalicylic acid and the Aminium salt, AED.

The produced red optical density measured at 545 nm was found to obey accurately Beer-Lambert law and the linearity extends to a greater absorbance value upto 3, Beer-Lambert plot for both of 3,5-dinitrosalicylic acid using Miller procedures and AED aminium salt has been shown in Fig. 1.

The sensitivity of this salt in reducing sugar determination is related to its intense color a unique property of all proton transfer complexes and/or salts [5,6] that is in turn definitely gives a reduced form intense color as well as its molecular structure has a four nitro groups. On the other hand, the reduced form color stability may be attributed to its good solubility without adding a color stabilizer.

It is noteworthy 3,5-dinitrosalicylic acid and ethylenediamine reaction afforded di proton transfer from both of carboxylic and hydroxyl groups into one molecule of ethylenediamine and it was found in a monomer form containing also two nitro groups [7] in contrary to this case of ethyl 3,5-dinitrosalicylate ester (1) and ethylenediamine reaction afforded dimeric form (2).

**Figure:**
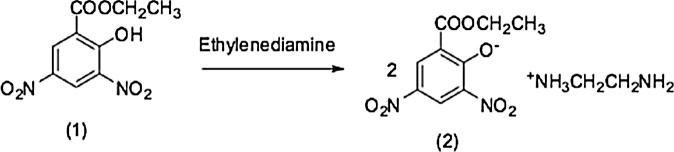

**Fig. 2:**
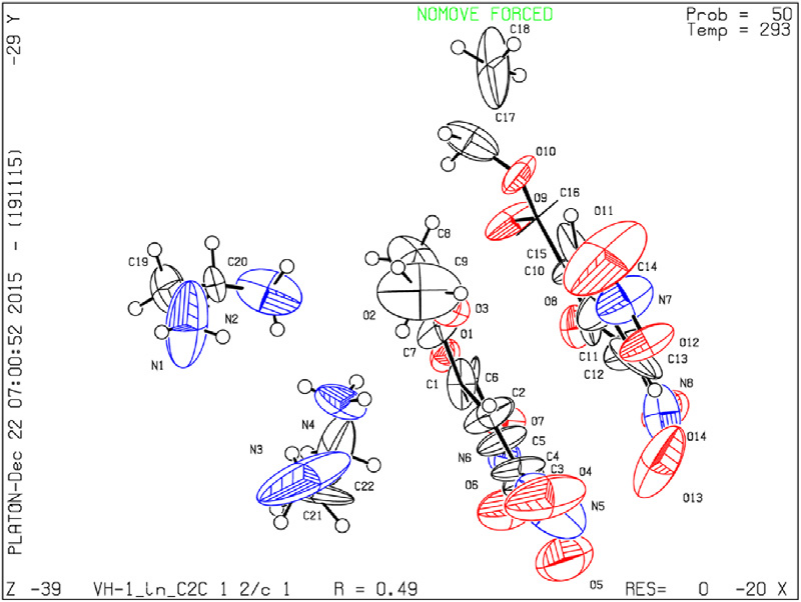
The molecular ORTEP diagram for 2-aminoethanaminium 2-(ethoxycarbonyl)-4,6-dinitrophenolate reveals it exists in the dimeric form.

The crystal structure analyses reveal it appears in the dimer form and it is crystallized in monoclinic crystal structure with centrosymmetric space group C2/c and the unit cell parameters are a = 15.289(2) Å, b = 20.755(3) Å and c = 20.545(3) Å. The crystal structure has been already deposited in Cambridge Crystallographic Data Centre coded CCDC 1441586, Fig. 2 represents the molecular ORTEP diagram for this aminium salt.

## Figures and Tables

**Figure:**
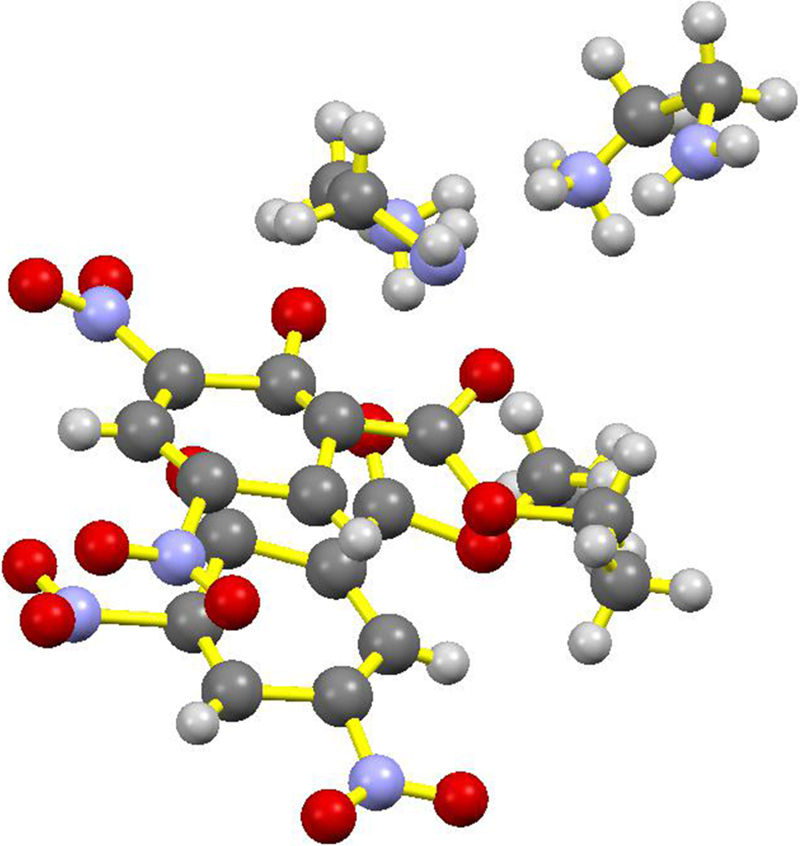


**Figure:**
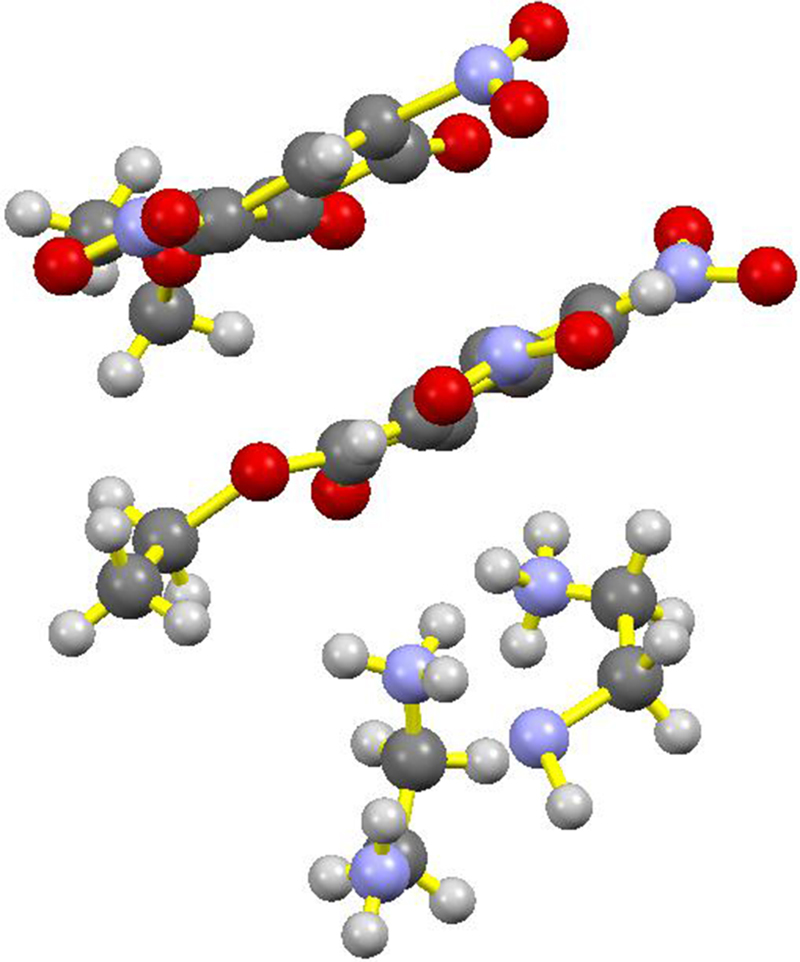


**Figure:**
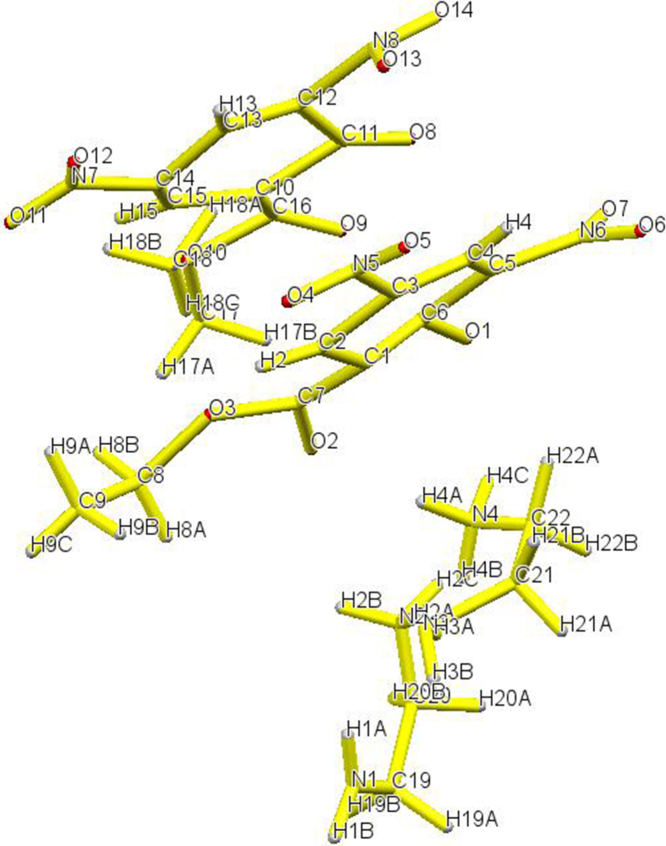


**Figure:**
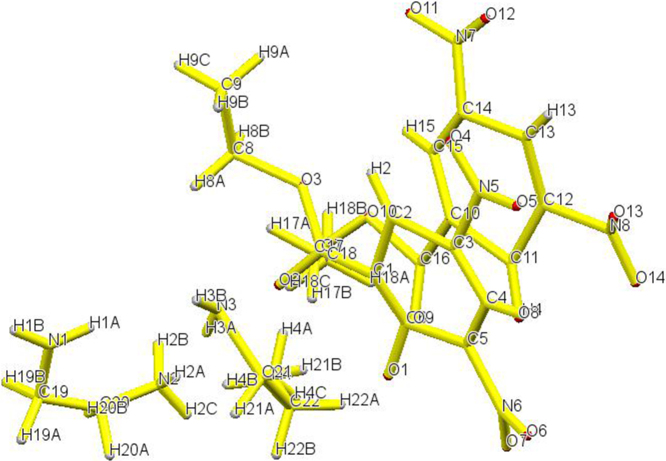


**Figure:**