# Supplementary material for: 2-Aminoethanaminium 2-(ethoxycarbonyl)-4,6-dinitrophenolate as a greener route in reducing sugar quantification
Source: MethodsX. 2018 Jun 5;5:609–12. doi: 10.1016/j.mex.2018.05.017 (PMC6008280; doi:10.1016/j.mex.2018.05.017)
Supplement: Supplementary file 3 [file mmc3.rtf]

X-ray Structure Report 


for 


VH-1_in_C2 


December 9, 2015 


Experimental 


Data Collection

	A yellow chip crystal of C22H33N8O14 having approximate dimensions of 0.500 x 0.300 x 0.300 mm was mounted on a glass fiber. All measurements were made on a Rigaku SCX mini diffractometer using graphite monochromated Mo-Ka radiation.

The crystal-to-detector distance was 52.00 mm.

	Cell constants and an orientation matrix for data collection corresponded to a C-centered monoclinic cell with dimensions:

           a  =   15.289(2) Å
           b  =   20.755(3) Å        b  =  100.283(3)o
           c  =   20.545(3) Å
           V  =  6415(2) Å3

For Z = 8 and F.W. = 633.55, the calculated density is 1.312 g/cm3. Based on the reflection conditions of:

           hkl:  h+k = 2n
           h0l:  l = 2n


packing considerations, a statistical analysis of intensity distribution, and the successful solution and refinement of the structure, the space group was determined to be:

C2/c (#15) 


	The data were collected at a temperature of 20 + 1oC to a maximum 2q value of 55.0o. A total of 540 oscillation images were collected. A sweep of data was done using w oscillations from -120.0 to 60.0o in 1.0o steps. The exposure rate was 10.0 [sec./o]. The detector swing angle was -30.80o.  A second sweep was performed using w oscillations from -120.0 to 60.0o in 1.0o steps. The exposure rate was 10.0 [sec./o]. The detector swing angle was -30.80o.  Another sweep was performed using w oscillations from -120.0 to 60.0o in 1.0o steps. The exposure rate was 10.0 [sec./o]. The detector swing angle was -30.80o.  The crystal-to-detector distance was 52.00 mm. Readout was performed in the 0.146 mm pixel mode.


Data Reduction

	Of the 32546 reflections that were collected, 7344 were unique (Rint = 0.0425); equivalent reflections were merged. Data were collected and processed using CrystalClear (Rigaku).

	The linear absorption coefficient, m, for Mo-Ka radiation is 1.103 cm-1. An empirical absorption correction was applied which resulted in transmission factors ranging from 0.762 to 0.967. The data were corrected for Lorentz and polarization effects. 


Structure Solution and Refinement

	The structure was solved by direct methods2 and expanded using Fourier techniques. The non-hydrogen atoms were refined anisotropically. Hydrogen atoms were refined using the riding model. The final cycle of full-matrix least-squares refinement3 on F2 was based on 7344 observed reflections and 405 variable parameters and converged (largest parameter shift was 1.31 times its esd) with unweighted and weighted agreement factors of: 

R1 = S ||Fo| - |Fc|| / S |Fo| = 0.4922 


 wR2 = [ S ( w (Fo2 - Fc2)2 )/ S w(Fo2)2]1/2 = 0.8498 


	The standard deviation of an observation of unit weight4 was 4.99. Unit weights were used.  The maximum and minimum peaks on the final difference Fourier map corresponded to 12.84 and -1.67 e/Å3, respectively. 

	Neutral atom scattering factors were taken from Cromer and Waber5. Anomalous dispersion effects were included in Fcalc6; the values for Df' and Df" were those of Creagh and McAuley7. The values for the mass attenuation coefficients are those of Creagh and Hubbell8. All calculations were performed using the CrystalStructure9 crystallographic software package except for refinement, which was performed using SHELXL-9710.


References 


(1) CrystalClear: Rigaku Corporation, 1999. CrystalClear Software User's Guide, Molecular Structure Corporation, (c) 2000.J.W.Pflugrath (1999) Acta Cryst. D55, 1718-1725.

(2) SIR92: Altomare, A., Cascarano, G., Giacovazzo, C., Guagliardi, A., Burla, M., Polidori, G., and Camalli, M. (1994) J. Appl. Cryst., 27, 435. 

(3) Least Squares function minimized: (SHELXL97)

		Sw(Fo2-Fc2)2     where w = Least Squares weights.

(4) Standard deviation of an observation of unit weight: 

 	 	 [Sw(Fo2-Fc2)2/(No-Nv)]1/2

	 	 where	 No  = number of observations
	 	 	 	 Nv  = number of variables 


(5) Cromer, D. T. & Waber, J. T.; "International Tables for X-ray Crystallography", Vol. IV, The Kynoch Press, Birmingham, England, Table 2.2 A (1974). 

(6) Ibers, J. A. & Hamilton, W. C.; Acta Crystallogr., 17, 781 (1964). 

(7) Creagh, D. C. & McAuley, W.J .; "International Tables for Crystallography", Vol C, (A.J.C. Wilson, ed.), Kluwer Academic Publishers, Boston, Table 4.2.6.8, pages 219-222 (1992). 

(8) Creagh, D. C. & Hubbell, J.H..; "International Tables for Crystallography", Vol C, (A.J.C. Wilson, ed.), Kluwer Academic Publishers, Boston, Table 4.2.4.3, pages 200-206 (1992). 

(9) CrystalStructure 4.0: Crystal Structure Analysis Package, Rigaku Corporation (2000-2010). Tokyo 196-8666, Japan.

(10) SHELX97: Sheldrick, G.M. (2008). Acta Cryst. A64, 112-122.


EXPERIMENTAL DETAILS 


A. Crystal Data 


Empirical Formula	C22H33N8O14

Formula Weight	633.55

Crystal Color, Habit	yellow, chip

Crystal Dimensions	0.500 X 0.300 X 0.300 mm

Crystal System	monoclinic

Lattice Type	C-centered

Lattice Parameters	a =  15.289(2) Å
	b =  20.755(3) Å
	c =  20.545(3) Å
	b = 100.283(3) o
	V = 6415(2) Å3

Space Group	C2/c (#15)

Z value	8

Dcalc	1.312 g/cm3

F000	2664.00

m(MoKa)	1.103 cm-1

B. Intensity Measurements 


Diffractometer	SCX mini

Radiation	MoKa (l = 0.71075 Å)
	graphite monochromated

Voltage, Current	50kV, 30mA

Temperature	20.0oC

Detector Aperture	75 mm (diameter)

Data Images	540 exposures

w oscillation Range 	-120.0 - 60.0o

Exposure Rate 	10.0 sec./o

Detector Swing Angle	-30.80o

w oscillation Range 	-120.0 - 60.0o

Exposure Rate 	10.0 sec./o

Detector Swing Angle	-30.80o

w oscillation Range 	-120.0 - 60.0o

Exposure Rate 	10.0 sec./o

Detector Swing Angle	-30.80o

Detector Position	52.00 mm

Pixel Size	0.146 mm

2qmax	55.0o

No. of Reflections Measured	Total: 32546
	Unique: 7344 (Rint = 0.0425)

Corrections	Lorentz-polarization
		Absorption
		(trans. factors: 0.762 - 0.967)

C. Structure Solution and Refinement 


Structure Solution	Direct Methods (SIR92)

Refinement	Full-matrix least-squares on F2

Function Minimized	S w (Fo2 - Fc2)2 

Least Squares Weights	w = 1/ [ s2(Fo2) + (0.2000 . P)2 
	 + 0.0000 .  P ]
	 where P = (Max(Fo2,0) + 2Fc2)/3

2qmax cutoff	55.0o

Anomalous Dispersion	All non-hydrogen atoms

No. Observations (All reflections)	7344

No. Variables	405

Reflection/Parameter Ratio	18.13

Residuals: R1 (I>2.00s(I))	0.4922

Residuals: R (All reflections)	0.5252

Residuals: wR2 (All reflections)	0.8498

Goodness of Fit Indicator	4.988

Max Shift/Error in Final Cycle	1.311

Maximum peak in Final Diff. Map	12.84 e/Å3

Minimum peak in Final Diff. Map	-1.67 e/Å3


Table 1. Atomic coordinates and Biso/Beq

atom	   x	   y	   z	 Beq
O1    	 0.7170(7)	 0.2132(5)	 0.5484(6)	 4.0(3)
O2    	 0.6693(8)	 0.3226(8)	 0.6096(7)	 5.6(3)
O3    	 0.675(2)	 0.3095(9)	 0.7113(7)	 6.7(4)
O4    	 0.614(3)	 0.103(2)	 0.800(2)	 16(2)
O5    	 0.616(3)	 0.022(2)	 0.759(2)	 16(2)
O6    	 0.687(2)	 0.025(1)	 0.5339(8)	 8.9(6)
O7    	 0.771(1)	 0.0971(7)	 0.5088(6)	 5.5(3)
O8    	 0.8975(9)	 0.1679(8)	 0.6177(6)	 5.6(3)
O9    	 0.907(2)	 0.2959(5)	 0.5834(6)	 7.1(5)
O10   	 0.910(1)	 0.3634(6)	 0.6708(7)	 6.3(4)
O11   	 0.856(4)	 0.303(2)	 0.881(2)	 19(2)
O12   	 0.841(2)	 0.2039(6)	 0.9008(8)	 6.3(4)
O13   	 0.843(3)	 0.035(2)	 0.748(2)	 14(1)
O14   	 0.928(2)	 0.049(1)	 0.680(1)	 9.7(6)
N1    	 0.377(3)	 0.421(3)	 0.524(2)	 15(2)
N2    	 0.536(2)	 0.381(1)	 0.498(2)	 8.8(7)
N3    	 0.380(7)	 0.229(3)	 0.597(3)	 26(5)
N4    	 0.530(2)	 0.242(2)	 0.5346(9)	 6.9(6)
N5    	 0.638(3)	 0.076(3)	 0.759(2)	 13(2)
N6    	 0.722(1)	 0.0775(8)	 0.547(1)	 5.6(4)
N7    	 0.858(3)	 0.246(2)	 0.868(1)	 9.4(7)
N8    	 0.895(2)	 0.069(2)	 0.725(1)	 7.2(5)
C1    	 0.6657(9)	 0.2102(9)	 0.6471(6)	 4.3(4)
C2    	 0.647(2)	 0.185(1)	 0.706(2)	 8.2(8)
C3    	 0.655(2)	 0.113(1)	 0.698(2)	 7.1(6)
C4    	 0.673(2)	 0.0801(9)	 0.6496(8)	 5.7(5)
C5    	 0.702(2)	 0.1141(7)	 0.601(1)	 6.1(5)
C6    	 0.7096(9)	 0.193(1)	 0.604(1)	 5.5(5)
C7    	 0.677(2)	 0.281(1)	 0.654(3)	 10(2)
C8    	 0.659(3)	 0.380(2)	 0.724(2)	 11(2)
C9    	 0.605(4)	 0.375(2)	 0.786(2)	 15(2)
C10   	 0.889(2)	 0.253(2)	 0.694(2)	 8.6(9)
C11   	 0.898(2)	 0.182(1)	 0.6701(9)	 6.0(6)
C12   	 0.896(3)	 0.149(2)	 0.731(1)	 8.3(8)
C13   	 0.866(2)	 0.159(2)	 0.795(3)	 14(2)
C14   	 0.864(3)	 0.233(2)	 0.802(3)	 12(2)
C15   	 0.889(2)	 0.282(2)	 0.7520(7)	 7.4(8)


Table 1. Atomic coordinates and Biso/Beq (continued)

atom	   x	   y	   z	 Beq
C16   	 0.916(3)	 0.3010(7)	 0.640(2)	 11(2)
C17   	 0.893(2)	 0.410(2)	 0.624(2)	 9(1)
C18   	 0.994(3)	 0.452(2)	 0.610(3)	 17(3)
C19   	 0.407(2)	 0.444(1)	 0.468(1)	 6.0(5)
C20   	 0.480(2)	 0.412(1)	 0.464(1)	 5.6(5)
C21   	 0.409(2)	 0.185(2)	 0.550(3)	 13(2)
C22   	 0.490(3)	 0.195(2)	 0.516(3)	 13(2)

Beq = 8/3 p2(U11(aa*)2 + U22(bb*)2 + U33(cc*)2 + 2U12(aa*bb*)cos g + 2U13(aa*cc*)cos b + 2U23(bb*cc*)cos a)


Table 2. Atomic coordinates and Biso involving hydrogen atoms

atom	   x	   y	   z	 Biso
H1A   	 0.4056	 0.3906	 0.5474	 17.90
H1B   	 0.3291	 0.4361	 0.5346	 17.90
H2    	 0.6319	 0.2069	 0.7419	 9.83
H2A   	 0.5117	 0.3468	 0.5135	 10.61
H2B   	 0.5636	 0.4049	 0.5312	 10.61
H2C   	 0.5757	 0.3678	 0.4734	 10.61
H3A   	 0.403(9)	 0.221(8)	 0.611(8)	 1(3)
H3B   	 0.298(7)	 0.209(5)	 0.613(5)	 0(2)
H4    	 0.6673	 0.0355	 0.6477	 6.85
H4A   	 0.5435	 0.2418	 0.5786	 8.29
H4B   	 0.4976	 0.2764	 0.5210	 8.29
H4C   	 0.5800	 0.2427	 0.5180	 8.29
H8A   	 0.6232	 0.4006	 0.6860	 12.94
H8B   	 0.7144	 0.4037	 0.7364	 12.94
H9A   	 0.6434	 0.3565	 0.8233	 18.13
H9B   	 0.5537	 0.3488	 0.7733	 18.13
H9C   	 0.5877	 0.4176	 0.7971	 18.13
H13   	 0.8514	 0.1285	 0.8243	 17.07
H15   	 0.9018	 0.3248	 0.7608	 8.85
H17A  	 0.8525	 0.4414	 0.6375	 11.21
H17B  	 0.8637	 0.3910	 0.5828	 11.21
H18A  	 1.0335	 0.4217	 0.5955	 19.99
H18B  	 1.0220	 0.4723	 0.6500	 19.99
H18C  	 0.9776	 0.4844	 0.5762	 19.99
H19A  	 0.3630	 0.4376	 0.4288	 7.20
H19B  	 0.4199	 0.4899	 0.4730	 7.20
H20A  	 0.4594	 0.3833	 0.4275	 6.66
H20B  	 0.5146	 0.4446	 0.4459	 6.66
H21A  	 0.3585	 0.1797	 0.5149	 15.55
H21B  	 0.4182	 0.1441	 0.5727	 15.55
H22A  	 0.5285	 0.1580	 0.5252	 15.64
H22B  	 0.4695	 0.1978	 0.4690	 15.64


Table 3. Anisotropic displacement parameters

atom	  U11	  U22	  U33	  U12	  U13	  U23
O1    	0.048(6)	0.054(6)	0.054(7)	 0.011(5)	 0.017(5)	 0.019(5)
O2    	0.053(7)	0.082(9)	0.083(9)	-0.027(7)	 0.024(6)	-0.039(8)
O3    	0.10(1)	0.10(2)	0.053(8)	-0.032(9)	 0.007(8)	 0.017(8)
O4    	0.39(6)	0.15(3)	0.08(2)	-0.12(3)	 0.06(3)	 0.01(2)
O5    	0.29(4)	0.10(2)	0.17(3)	-0.07(2)	-0.08(3)	 0.03(2)
O6    	0.19(2)	0.10(2)	0.062(9)	-0.05(2)	 0.05(1)	 -0.008(9)
O7    	0.088(9)	0.068(8)	0.059(8)	-0.028(7)	 0.027(7)	-0.012(6)
O8    	0.070(8)	0.09(1)	0.044(7)	 0.008(7)	 -0.004(6)	 0.007(7)
O9    	0.21(2)	0.026(6)	0.026(6)	-0.009(7)	 0.003(8)	-0.016(4)
O10   	0.13(2)	0.033(6)	0.064(8)	 0.005(7)	-0.012(8)	 0.021(6)
O11   	0.49(8)	0.08(2)	0.12(2)	 0.01(3)	 -0.03(3)	-0.05(2)
O12   	0.14(2)	0.057(8)	0.064(9)	 -0.001(7)	 0.061(9)	 -0.001(6)
O13   	0.21(3)	0.16(3)	0.17(3)	 0.10(3)	 0.06(3)	 0.09(2)
O14   	0.17(2)	0.09(2)	0.10(2)	-0.02(2)	 0.00(2)	 0.03(1)
N1    	0.18(3)	0.30(6)	0.11(2)	 0.11(3)	 0.08(2)	 0.11(3)
N2    	0.11(2)	0.10(2)	0.13(2)	 0.01(2)	 0.04(2)	-0.07(2)
N3    	0.6(2)	0.14(4)	0.18(5)	-0.13(6)	-0.19(7)	-0.04(3)
N4    	0.07(2)	0.13(2)	0.06(1)	-0.05(2)	 0.019(8)	 0.01(1)
N5    	0.19(3)	0.26(5)	0.06(2)	-0.07(3)	 0.06(2)	-0.07(2)
N6    	0.048(8)	0.051(9)	0.11(2)	 0.027(7)	 0.011(9)	 -0.000(9)
N7    	0.20(3)	0.11(2)	0.06(2)	 -0.01(2)	 0.04(2)	 0.00(2)
N8    	0.09(2)	0.12(2)	0.06(1)	 0.04(2)	 0.01(1)	-0.01(2)
C1    	0.044(7)	0.12(2)	0.009(5)	 0.038(8)	 0.036(5)	 0.017(6)
C2    	0.09(2)	0.06(2)	0.18(3)	 0.02(1)	 0.09(2)	 0.05(2)
C3    	0.11(2)	0.05(1)	0.11(2)	-0.04(1)	 0.05(2)	 0.03(2)
C4    	0.13(2)	0.05(1)	0.035(8)	-0.02(1)	 0.00(1)	 0.013(7)
C5    	0.14(2)	0.018(7)	0.08(2)	-0.015(9)	 0.04(2)	-0.018(7)
C6    	0.021(6)	0.09(2)	0.09(2)	 0.028(7)	 0.017(8)	 0.07(1)
C7    	0.11(2)	0.05(1)	0.23(4)	 0.05(2)	 0.12(3)	 0.05(2)
C8    	0.10(2)	0.15(3)	0.15(3)	 -0.00(2)	 0.00(2)	-0.10(3)
C9    	0.35(7)	0.13(3)	0.14(3)	 -0.01(4)	 0.17(5)	-0.04(3)
C10   	0.08(2)	0.09(2)	0.14(2)	-0.03(2)	-0.03(2)	 0.11(2)
C11   	0.07(1)	0.07(1)	0.07(1)	 0.06(1)	-0.030(9)	-0.042(9)
C12   	0.20(3)	0.08(2)	0.05(1)	-0.03(2)	 0.07(2)	 0.01(1)
C13   	0.07(2)	0.09(2)	0.34(7)	-0.02(2)	-0.06(3)	-0.11(3)
C14   	0.17(4)	0.12(3)	0.15(4)	 -0.01(3)	 -0.01(3)	 0.09(3)
C15   	0.048(9)	0.22(3)	0.002(5)	 0.02(2)	-0.016(6)	-0.04(1)


Table 3. Anisotropic displacement parameters (continued)

atom	  U11	  U22	  U33	  U12	  U13	  U23
C16   	0.27(4)	0.008(6)	0.09(2)	-0.04(1)	-0.12(2)	 0.036(8)
C17   	0.12(2)	0.13(3)	0.12(3)	-0.05(2)	 0.05(2)	-0.10(2)
C18   	0.16(3)	0.15(3)	0.26(5)	 0.12(3)	-0.12(4)	-0.14(4)
C19   	0.05(1)	0.09(2)	0.09(2)	 0.03(1)	 0.007(9)	-0.01(1)
C20   	0.05(1)	0.07(2)	0.08(2)	 -0.004(9)	-0.024(9)	 0.02(1)
C21   	0.10(2)	0.11(2)	0.33(5)	-0.09(2)	 0.16(3)	-0.12(3)
C22   	0.09(3)	0.17(4)	0.26(5)	 0.10(3)	 0.10(3)	 0.10(4)


The general temperature factor expression: exp(-2p2(a*2U11h2 + b*2U22k2 + c*2U33l2 + 2a*b*U12hk + 2a*c*U13hl + 2b*c*U23kl))


Table 4. Fragment Analysis


fragment: 1  
	O(1)	O(2)	O(3)	O(4)	O(5)
	O(6)	O(7)	N(5)	N(6)	C(1)
	C(2)	C(3)	C(4)	C(5)	C(6)
	C(7)	C(8)	C(9)


fragment: 2  
	O(8)	O(9)	O(10)	O(11)	O(12)
	O(13)	O(14)	N(7)	N(8)	C(10)
	C(11)	C(12)	C(13)	C(14)	C(15)
	C(16)	C(17)	C(18)


fragment: 3  
	N(1)	N(2)	C(19)	C(20)


fragment: 4  
	N(3)	N(4)	C(21)	C(22)


Table 5. Bond lengths (Å)

atom	atom	distance		atom	atom	distance
O1	C6	1.24(3)		O2	C7	1.25(4)	
O3	C7	1.32(5)		O3	C8	1.52(5)	
O4	N5	1.13(5)		O5	N5	1.17(6)	
O6	N6	1.21(3)		O7	N6	1.24(3)	
O8	C11	1.12(3)		O9	C16	1.16(3)	
O10	C16	1.45(2)		O10	C17	1.36(4)	
O11	N7	1.20(4)		O12	N7	1.16(3)	
O13	N8	1.21(5)		O14	N8	1.22(4)	
N1	C19	1.40(5)		N2	C20	1.20(3)	
N3	C21	1.44(8)		N4	C22	1.18(5)	
N5	C3	1.52(5)		N6	C5	1.43(3)	
N7	C14	1.41(6)		N8	C12	1.66(4)	
C1	C2	1.40(4)		C1	C6	1.26(3)	
C1	C7	1.48(3)		C2	C3	1.51(3)	
C3	C4	1.28(4)		C4	C5	1.35(3)	
C5	C6	1.63(3)		C8	C9	1.63(7)	
C10	C11	1.56(4)		C10	C15	1.33(4)	
C10	C16	1.60(4)		C11	C12	1.44(3)	
C12	C13	1.48(6)		C13	C14	1.54(6)	
C14	C15	1.53(5)		C17	C18	1.84(5)	
C19	C20	1.30(3)		C21	C22	1.53(6)	


Table 6. Bond lengths involving hydrogens (Å)

atom	atom	distance		atom	atom	distance
N1	H1A	0.860		N1	H1B	0.860	
N2	H2A	0.890		N2	H2B	0.890	
N2	H2C	0.890		N3	H3A	0.44(15)	
N3	H3B	1.41(14)		N4	H4A	0.890	
N4	H4B	0.890		N4	H4C	0.890	
C2	H2	0.930		C4	H4	0.930	
C8	H8A	0.970		C8	H8B	0.970	
C9	H9A	0.960		C9	H9B	0.960	
C9	H9C	0.960		C13	H13	0.930	
C15	H15	0.930		C17	H17A	0.970	
C17	H17B	0.970		C18	H18A	0.960	
C18	H18B	0.960		C18	H18C	0.960	
C19	H19A	0.970		C19	H19B	0.970	
C20	H20A	0.970		C20	H20B	0.970	
C21	H3A	1.47(16)		C21	H21A	0.970	
C21	H21B	0.970		C22	H22A	0.970	
C22	H22B	0.970		


Table 7. Bond angles (o)

atom	atom	atom	angle		atom	atom	atom	angle
C7	O3	C8	128(3)		C16	O10	C17	110.5(18)
O4	N5	O5	110(4)		O4	N5	C3	120(4)
O5	N5	C3	125(3)		O6	N6	O7	116.2(19)
O6	N6	C5	119.7(19)		O7	N6	C5	124.0(16)
O11	N7	O12	126(3)		O11	N7	C14	114(3)
O12	N7	C14	118(3)		O13	N8	O14	119(3)
O13	N8	C12	124(3)		O14	N8	C12	113(3)
C2	C1	C6	136.0(19)		C2	C1	C7	109(3)
C6	C1	C7	107(3)		C1	C2	C3	104(3)
N5	C3	C2	112(3)		N5	C3	C4	118(3)
C2	C3	C4	130(3)		C3	C4	C5	115.9(19)
N6	C5	C4	116.2(15)		N6	C5	C6	121.8(18)
C4	C5	C6	122.0(18)		O1	C6	C1	133.9(18)
O1	C6	C5	109.5(17)		C1	C6	C5	105.5(18)
O2	C7	O3	109(2)		O2	C7	C1	128(4)
O3	C7	C1	121(3)		O3	C8	C9	101(3)
C11	C10	C15	136(3)		C11	C10	C16	109(2)
C15	C10	C16	112(2)		O8	C11	C10	124(2)
O8	C11	C12	137(2)		C10	C11	C12	99.1(19)
N8	C12	C11	114.1(19)		N8	C12	C13	102(3)
C11	C12	C13	140(3)		C12	C13	C14	104(4)
N7	C14	C13	107(4)		N7	C14	C15	126(4)
C13	C14	C15	126(4)		C10	C15	C14	110(3)
O9	C16	O10	120.9(15)		O9	C16	C10	129.8(19)
O10	C16	C10	102(2)		O10	C17	C18	113(3)
N1	C19	C20	107(3)		N2	C20	C19	140(3)
N3	C21	C22	126(5)		N4	C22	C21	113(4)


Table 8. Bond angles involving hydrogens (o)

atom	atom	atom	angle		atom	atom	atom	angle
C19	N1	H1A	120.0		C19	N1	H1B	120.0
H1A	N1	H1B	120.0		C20	N2	H2A	109.5
C20	N2	H2B	109.5		C20	N2	H2C	109.5
H2A	N2	H2B	109.5		H2A	N2	H2C	109.5
H2B	N2	H2C	109.5		C21	N3	H3A	84(23)
C21	N3	H3B	111(6)		H3A	N3	H3B	112(23)
C22	N4	H4A	109.5		C22	N4	H4B	109.5
C22	N4	H4C	109.5		H4A	N4	H4B	109.5
H4A	N4	H4C	109.5		H4B	N4	H4C	109.5
C1	C2	H2	127.9		C3	C2	H2	127.9
C3	C4	H4	122.1		C5	C4	H4	122.0
O3	C8	H8A	111.5		O3	C8	H8B	111.5
C9	C8	H8A	111.5		C9	C8	H8B	111.5
H8A	C8	H8B	109.3		C8	C9	H9A	109.5
C8	C9	H9B	109.5		C8	C9	H9C	109.5
H9A	C9	H9B	109.5		H9A	C9	H9C	109.5
H9B	C9	H9C	109.5		C12	C13	H13	128.1
C14	C13	H13	128.0		C10	C15	H15	125.0
C14	C15	H15	125.0		O10	C17	H17A	108.9
O10	C17	H17B	108.9		C18	C17	H17A	108.9
C18	C17	H17B	108.9		H17A	C17	H17B	107.8
C17	C18	H18A	109.5		C17	C18	H18B	109.5
C17	C18	H18C	109.5		H18A	C18	H18B	109.5
H18A	C18	H18C	109.5		H18B	C18	H18C	109.5
N1	C19	H19A	110.4		N1	C19	H19B	110.4
C20	C19	H19A	110.4		C20	C19	H19B	110.4
H19A	C19	H19B	108.6		N2	C20	H20A	101.9
N2	C20	H20B	102.0		C19	C20	H20A	102.0
C19	C20	H20B	102.0		H20A	C20	H20B	104.7
N3	C21	H3A	17(6)		N3	C21	H21A	105.9
N3	C21	H21B	105.8		C22	C21	H3A	120(6)
C22	C21	H21A	105.9		C22	C21	H21B	105.9
H3A	C21	H21A	122.3		H3A	C21	H21B	94.0
H21A	C21	H21B	106.2		N4	C22	H22A	109.0
N4	C22	H22B	109.0		C21	C22	H22A	109.0
C21	C22	H22B	109.0		H22A	C22	H22B	107.8
N3	H3A	C21	78(21)


Table 9. Torsion Angles(o)
	(Those having bond angles > 160 or < 20 degrees are excluded.)

atom1	atom2	atom3	atom4	   angle		atom1	atom2	atom3	atom4	   angle
C7	O3	C8	C9	145(3) 		C8	O3	C7	O2	5(4) 
C8	O3	C7	C1	-161(3) 		C16	O10	C17	C18	-96(3) 
C17	O10	C16	O9	-1(4) 		C17	O10	C16	C10	-153.8(19) 
O4	N5	C3	C2	7(5) 		O4	N5	C3	C4	-173(4) 
O5	N5	C3	C2	157(4) 		O5	N5	C3	C4	-22(5) 
O6	N6	C5	C4	25(3) 		O6	N6	C5	C6	-153.7(18) 
O7	N6	C5	C4	-159.1(16) 		O7	N6	C5	C6	22(3) 
O11	N7	C14	C13	-179(4) 		O11	N7	C14	C15	15(6) 
O12	N7	C14	C13	-12(5) 		O12	N7	C14	C15	-178(3) 
O13	N8	C12	C11	-131(3) 		O13	N8	C12	C13	31(4) 
O14	N8	C12	C11	27(4) 		O14	N8	C12	C13	-171(2) 
C2	C1	C6	O1	170(2) 		C2	C1	C6	C5	32(3) 
C6	C1	C2	C3	-25(3) 		C2	C1	C7	O2	-158(3) 
C2	C1	C7	O3	5(3) 		C7	C1	C2	C3	-168.8(16) 
C6	C1	C7	O2	48(4) 		C6	C1	C7	O3	-149(2) 
C7	C1	C6	O1	-46(3) 		C7	C1	C6	C5	176.0(16) 
C1	C2	C3	N5	179.0(16) 		C1	C2	C3	C4	-2(4) 
N5	C3	C4	C5	-170(2) 		C2	C3	C4	C5	11(4) 
C3	C4	C5	N6	180(2) 		C3	C4	C5	C6	-2(4) 
N6	C5	C6	O1	13(3) 		N6	C5	C6	C1	163.0(17) 
C4	C5	C6	O1	-164.9(19) 		C4	C5	C6	C1	-15(3) 
C11	C10	C15	C14	-21(4) 		C15	C10	C11	O8	-179(3) 
C15	C10	C11	C12	6(3) 		C11	C10	C16	O9	33(4) 
C11	C10	C16	O10	-177.1(17) 		C16	C10	C11	O8	-21(3) 
C16	C10	C11	C12	164.2(18) 		C15	C10	C16	O9	-163(3) 
C15	C10	C16	O10	-14(3) 		C16	C10	C15	C14	-178.7(18) 
O8	C11	C12	N8	1(5) 		O8	C11	C12	C13	-152(3) 
C10	C11	C12	N8	175(2) 		C10	C11	C12	C13	22(4) 
N8	C12	C13	C14	-178.8(18) 		C11	C12	C13	C14	-24(5) 
C12	C13	C14	N7	-165(3) 		C12	C13	C14	C15	0(5) 
N7	C14	C15	C10	179(4) 		C13	C14	C15	C10	17(4) 
N1	C19	C20	N2	-21(4) 		N3	C21	C22	N4	3(6) 


Table 10. Possible hydrogen bonds

Donor	 H	Acceptor	 D...A	D-H	H...A	  D-H...A
 N1	 H1A	  N2		2.72(5)	0.86	2.41	  101.69  	intramol.
 N2	 H2A	  N1		2.72(5)	0.89	2.61	  87.46  	intramol.
 N2	 H2A	  N4		2.99(4)	0.89	2.23	  143.23  
 N2	 H2C	  O71		3.01(3)	0.89	2.42	  123.92  
 N2	 H2C	  O81		2.92(3)	0.89	2.12	  149.07  
 N4	 H4A	  O2		2.92(3)	0.89	2.55	  106.24  
 N4	 H4A	  N3		2.84(10)	0.89	2.61	  95.40  	intramol.
 N4	 H4B	  N2		2.99(4)	0.89	2.32	  132.08  
 N4	 H4C	  O1		2.88(3)	0.89	2.16	  137.25  
 N4	 H4C	  O91		2.87(3)	0.89	2.27	  124.45  


Symmetry Operators:

(1)  -X+1/2+1,-Y+1/2,-Z+1


Table 11. Intramolecular contacts less than 3.60 Å

atom	atom	distance		atom	atom	distance
O1	O2	2.756(19)		O1	O7	2.717(18)	
O1	N6	2.82(2)		O1	C4	3.59(3)	
O1	C7	2.75(5)		O2	C2	3.53(4)	
O2	C6	2.78(3)		O2	C8	2.67(5)	
O3	C2	2.63(3)		O3	C6	3.38(3)	
O4	C2	2.68(5)		O4	C4	3.41(4)	
O5	C2	3.59(4)		O5	C4	2.82(4)	
O6	C4	2.68(3)		O7	C4	3.50(3)	
O7	C6	3.04(3)		O8	O9	2.758(19)	
O8	O14	2.78(3)		O8	N8	3.02(3)	
O8	C16	2.81(2)		O9	C11	2.98(3)	
O9	C15	3.537(19)		O9	C17	2.54(4)	
O9	C18	3.51(4)		O10	C15	2.44(3)	
O11	C13	3.47(5)		O11	C15	2.81(4)	
O12	C13	2.45(6)		O13	C13	2.76(5)	
O14	C11	2.80(3)		O14	C13	3.55(6)	
N1	N2	2.72(5)		N3	N4	2.84(10)	
N6	C3	3.52(4)		N7	C12	3.59(4)	
C1	C4	2.70(3)		C2	C5	2.85(4)	
C3	C6	2.78(4)		C10	C13	2.91(6)	
C10	C17	3.57(4)		C11	C14	3.04(5)	
C12	C15	2.79(4)		C16	C18	3.45(5)	


Table 12. Intramolecular contacts less than 3.60 Å involving hydrogens

atom	atom	distance		atom	atom	distance
O2	H8A	2.443		O2	H8B	3.074	
O3	H2	2.351		O3	H9A	2.625	
O3	H9B	2.570		O3	H9C	3.284	
O4	H2	2.500		O5	H4	2.562	
O6	H4	2.420		O9	H17A	3.374	
O9	H17B	2.082		O9	H18A	3.231	
O10	H15	2.038		O10	H18A	2.912	
O10	H18B	2.915		O10	H18C	3.447	
O11	H15	2.718		O12	H13	2.242	
O13	H13	2.493		N1	H2A	2.609	
N1	H2B	2.853		N1	H2C	3.558	
N1	H20A	2.648		N1	H20B	2.910	
N2	H1A	2.411		N2	H1B	3.575	
N2	H19A	3.013		N2	H19B	2.867	
N3	H4A	2.610		N3	H4B	2.765	
N3	H22A	3.270		N3	H22B	3.237	
N4	H3A	2.74(16)		N4	H21A	2.888	
N4	H21B	2.853		N5	H2	2.728	
N5	H4	2.550		N6	H4	2.524	
N7	H13	2.598		N7	H15	2.915	
N8	H13	2.567		C2	H4	3.355	
C4	H2	3.368		C6	H2	3.278	
C6	H4	3.473		C7	H2	2.555	
C7	H8A	2.729		C7	H8B	3.051	
C11	H13	3.542		C11	H15	3.499	
C13	H15	3.569		C15	H13	3.597	
C16	H15	2.576		C16	H17A	3.071	
C16	H17B	2.279		C16	H18A	3.303	
C17	H15	3.305		C19	H2A	2.638	
C19	H2B	2.635		C19	H2C	3.008	
C20	H1A	2.263		C20	H1B	2.974	
C21	H4A	2.348		C21	H4B	2.463	
C21	H4C	3.052		H1A	H2A	2.085	
H1A	H2B	2.515		H1A	H2C	3.273	
H1A	H19A	2.599		H1A	H19B	2.599	
H1A	H20A	2.738		H1A	H20B	3.103	
H1B	H2A	3.442		H1B	H19A	2.324	


Table 12. Intramolecular contacts less than 3.60 Å involving hydrogens (continued)

atom	atom	distance		atom	atom	distance
H1B	H19B	2.324		H1B	H20A	3.402	
H2	H9A	3.517		H2	H9B	3.287	
H2A	H19A	3.215		H2A	H19B	3.326	
H2A	H20A	1.956		H2A	H20B	2.463	
H2B	H19A	3.464		H2B	H19B	2.905	
H2B	H20A	2.464		H2B	H20B	1.961	
H2C	H19A	3.529		H2C	H19B	3.477	
H2C	H20A	1.887		H2C	H20B	1.882	
H3A	H4A	2.400		H3A	H4B	2.789	
H3A	H22A	3.117		H3A	H22B	3.291	
H3B	H21A	2.431		H3B	H21B	2.522	
H4A	H21A	3.171		H4A	H21B	2.778	
H4A	H22A	2.048		H4A	H22B	2.505	
H4B	H21A	2.912		H4B	H21B	3.258	
H4B	H22A	2.503		H4B	H22B	1.957	
H4C	H21B	3.546		H4C	H22A	1.943	
H4C	H22B	2.032		H8A	H9A	2.929	
H8A	H9B	2.487		H8A	H9C	2.465	
H8B	H9A	2.453		H8B	H9B	2.928	
H8B	H9C	2.500		H15	H17A	3.486	
H17A	H18A	3.069		H17A	H18B	2.638	
H17A	H18C	2.628		H17B	H18A	2.640	
H17B	H18B	3.069		H17B	H18C	2.625	
H19A	H20A	1.859		H19A	H20B	2.286	
H19B	H20A	2.518		H19B	H20B	1.893	
H21A	H22A	2.609		H21A	H22B	2.116	
H21B	H22A	2.114		H21B	H22B	2.644	


Table 13. Intermolecular contacts less than 3.60 Å

atom	atom	distance		atom	atom	distance
O1	O11	2.835(17)		O1	O8	3.020(16)	
O1	O9	3.34(3)		O1	O91	3.022(17)	
O1	N4	2.88(3)		O1	C11	3.44(2)	
O1	C22	3.44(4)		O2	O71	3.21(2)	
O2	N2	3.03(3)		O2	N4	2.92(3)	
O3	C10	3.55(3)		O3	C14	3.52(5)	
O3	C15	3.27(3)		O4	N32	3.34(6)	
O4	C212	3.58(6)		O5	O52	3.51(6)	
O5	O13	3.53(6)		O5	C173	3.37(5)	
O6	C171	3.52(4)		O7	O21	3.21(2)	
O7	O8	3.063(18)		O7	N21	3.01(3)	
O7	C171	3.36(3)		O8	O1	3.020(16)	
O8	O7	3.063(18)		O8	N21	2.92(3)	
O8	N6	3.38(2)		O8	C5	3.16(3)	
O8	C6	2.881(19)		O8	C201	3.20(3)	
O9	O1	3.34(3)		O9	O11	3.022(17)	
O9	O114	3.57(7)		O9	N41	2.87(3)	
O9	C221	2.81(6)		O11	O94	3.57(7)	
O11	C164	3.58(8)		O11	C215	3.44(6)	
O11	C225	3.15(6)		O12	N15	3.59(5)	
O12	N32	3.43(10)		O12	C195	3.45(3)	
O12	C205	3.32(3)		O13	O5	3.53(6)	
O13	O144	3.55(5)		O13	N5	3.30(6)	
O13	C3	3.30(5)		O13	C4	3.14(4)	
O13	C83	3.26(5)		O13	C93	3.50(6)	
O14	O134	3.55(5)		O14	O144	3.30(3)	
O14	N84	3.07(3)		O14	C201	3.58(4)	
N1	O126	3.59(5)		N2	O2	3.03(3)	
N2	O71	3.01(3)		N2	O81	2.92(3)	
N2	N4	2.99(4)		N3	O42	3.34(6)	
N3	O122	3.43(10)		N4	O1	2.88(3)	
N4	O2	2.92(3)		N4	O91	2.87(3)	
N4	N2	2.99(4)		N4	C1	2.89(2)	
N4	C6	3.03(3)		N4	C7	3.13(4)	
N5	O13	3.30(6)		N6	O8	3.38(2)	
N7	C225	3.55(5)		N8	O144	3.07(3)	
N8	N84	3.20(3)		N8	C4	3.47(3)	


Table 13. Intermolecular contacts less than 3.60 Å (continued)

atom	atom	distance		atom	atom	distance
N8	C124	3.58(4)		C1	N4	2.89(2)	
C1	C10	3.49(3)		C1	C11	3.54(3)	
C1	C22	3.46(5)		C2	C13	3.56(4)	
C3	O13	3.30(5)		C4	O13	3.14(4)	
C4	N8	3.47(3)		C5	O8	3.16(3)	
C5	C11	3.38(3)		C6	O8	2.881(19)	
C6	N4	3.03(3)		C6	C10	3.27(3)	
C6	C11	2.96(3)		C6	C22	3.52(4)	
C7	N4	3.13(4)		C7	C10	3.25(4)	
C7	C15	3.49(4)		C8	O137	3.26(5)	
C9	O137	3.50(6)		C9	C92	3.30(8)	
C10	O3	3.55(3)		C10	C1	3.49(3)	
C10	C6	3.27(3)		C10	C7	3.25(4)	
C10	C154	3.43(3)		C11	O1	3.44(2)	
C11	C1	3.54(3)		C11	C5	3.38(3)	
C11	C6	2.96(3)		C11	C124	3.50(4)	
C11	C134	3.59(4)		C12	N84	3.58(4)	
C12	C114	3.50(4)		C12	C124	3.14(5)	
C13	C2	3.56(4)		C13	C114	3.59(4)	
C14	O3	3.52(5)		C15	O3	3.27(3)	
C15	C7	3.49(4)		C15	C104	3.43(3)	
C15	C154	3.41(3)		C15	C164	3.40(4)	
C16	O114	3.58(8)		C16	C154	3.40(4)	
C17	O57	3.37(5)		C17	O61	3.52(4)	
C17	O71	3.36(3)		C19	O126	3.45(3)	
C19	C208	3.59(3)		C20	O81	3.20(3)	
C20	O126	3.32(3)		C20	O141	3.58(4)	
C20	C198	3.59(3)		C21	O42	3.58(6)	
C21	O116	3.44(6)		C22	O1	3.44(4)	
C22	O91	2.81(6)		C22	O116	3.15(6)	
C22	N76	3.55(5)		C22	C1	3.46(5)	
C22	C6	3.52(4)		


Symmetry Operators:

(1)  -X+1/2+1,-Y+1/2,-Z+1		(2)  -X+1,Y,-Z+1/2+1
(3)  -X+1/2+1,Y+1/2-1,-Z+1/2+1		(4)  -X+2,Y,-Z+1/2+1
(5)  X+1/2,-Y+1/2,Z+1/2		(6)  X+1/2-1,-Y+1/2,Z+1/2-1
(7)  -X+1/2+1,Y+1/2,-Z+1/2+1		(8)  -X+1,-Y+1,-Z+1


Table 14. Intermolecular contacts less than 3.60 Å involving hydrogens

atom	atom	distance		atom	atom	distance
O1	H4A	2.890		O1	H4B	3.551	
O1	H4C	2.164		O1	H17B1	3.504	
O1	H22A	3.058		O2	H2A	2.874	
O2	H2B	2.681		O2	H2C	3.053	
O2	H4A	2.545		O2	H4B	3.068	
O2	H4C	2.691		O2	H17B	3.426	
O3	H4A	3.393		O3	H15	3.443	
O4	H3A2	3.09(16)		O4	H3B2	2.98(10)	
O4	H21B2	2.865		O5	H8B3	3.564	
O5	H17A3	2.684		O5	H18B4	2.653	
O5	H18B3	3.232		O6	H1B5	2.851	
O6	H17A1	3.533		O6	H17B1	2.949	
O6	H18A4	3.580		O6	H18C4	3.569	
O6	H18C1	3.077		O7	H1B5	3.474	
O7	H2B1	2.800		O7	H2C1	2.424	
O7	H17A1	3.345		O7	H17B1	2.537	
O7	H19B5	3.360		O7	H20B1	3.362	
O8	H2A1	3.258		O8	H2B1	3.556	
O8	H2C1	2.120		O8	H4C1	3.416	
O8	H20A1	2.739		O8	H20B1	3.096	
O9	H4B1	3.176		O9	H4C1	2.270	
O9	H22A1	2.765		O9	H22B1	2.336	
O10	H8B	3.589		O10	H156	3.076	
O11	H3B2	3.08(11)		O11	H9A	3.437	
O11	H18A6	2.988		O11	H21A7	2.775	
O11	H22B7	2.280		O12	H1A7	3.582	
O12	H2A7	3.330		O12	H3B2	2.11(10)	
O12	H4B7	3.141		O12	H19A7	2.999	
O12	H20A7	2.543		O12	H21A7	3.344	
O12	H22B7	2.998		O13	H4	3.080	
O13	H8A3	3.104		O13	H8B3	2.897	
O13	H9C3	2.863		O14	H2C1	3.576	
O14	H9C3	2.789		O14	H20A1	3.331	
O14	H20B1	2.866		N1	H4B	3.523	
N1	H9A2	3.474		N1	H20B8	3.256	
N2	H4A	3.324		N2	H4B	2.321	
N2	H4C	2.959		N2	H19B8	2.801	


Table 14. Intermolecular contacts less than 3.60 Å involving hydrogens (continued)

atom	atom	distance		atom	atom	distance
N3	H1A	3.548		N3	H22	3.381	
N3	H9A2	3.169		N4	H2A	2.230	
N4	H2B	3.425		N4	H2C	3.036	
N5	H13	3.466		N5	H17A3	3.508	
N5	H18B4	3.380		N6	H1B5	3.392	
N6	H2C1	3.389		N6	H17B1	2.827	
N6	H22A	3.356		N7	H3B2	2.62(11)	
N7	H4B7	3.497		N7	H20A7	3.226	
N7	H21A7	3.384		N7	H22B7	2.698	
N8	H9C3	3.192		C1	H4A	2.228	
C1	H4B	3.582		C1	H4C	2.823	
C1	H22A	3.158		C2	H4A	3.045	
C3	H18B4	3.595		C4	H18B4	3.219	
C4	H22A	3.468		C5	H4A	3.561	
C5	H4C	3.518		C5	H22A	2.967	
C6	H4A	2.700		C6	H4C	2.621	
C6	H22A	3.032		C7	H4A	2.472	
C7	H4B	3.513		C7	H4C	3.032	
C8	H9B2	3.326		C9	H1A2	3.475	
C9	H9B2	2.577		C9	H9C2	3.256	
C10	H156	3.502		C11	H2C1	3.219	
C11	H20A1	3.493		C13	H3B2	3.55(12)	
C13	H19A7	3.411		C13	H20A7	2.973	
C14	H2	3.586		C14	H3B2	3.33(12)	
C15	H156	3.379		C16	H4C1	3.382	
C16	H156	3.176		C16	H22B1	3.078	
C18	H49	3.150		C18	H22A1	3.562	
C18	H22B1	3.597		C19	H2B8	3.163	
C19	H1310	3.295		C19	H19B8	3.027	
C19	H20B8	3.019		C20	H4B	3.048	
C20	H1310	3.287		C20	H19B8	2.726	
C20	H20B8	3.492		C22	H2A	3.169	
C22	H18A1	3.317		H1A	O1210	3.582	
H1A	N3	3.548		H1A	C92	3.475	
H1A	H4B	2.857		H1A	H9A2	2.971	
H1A	H9C2	3.227		H1B	O611	2.851	
H1B	O711	3.474		H1B	N611	3.392	


Table 14. Intermolecular contacts less than 3.60 Å involving hydrogens (continued)

atom	atom	distance		atom	atom	distance
H1B	H9A2	3.316		H1B	H9C2	3.486	
H1B	H20B8	3.414		H2	N32	3.381	
H2	C14	3.586		H2	H3A2	3.177	
H2	H3B2	2.987		H2	H4A	3.462	
H2A	O2	2.874		H2A	O81	3.258	
H2A	O1210	3.330		H2A	N4	2.230	
H2A	C22	3.169		H2A	H4A	2.559	
H2A	H4B	1.489		H2A	H4C	2.396	
H2A	H19B8	3.542		H2A	H22B	3.258	
H2B	O2	2.681		H2B	O71	2.800	
H2B	O81	3.556		H2B	N4	3.425	
H2B	C198	3.163		H2B	H4A	3.550	
H2B	H4B	2.845		H2B	H4C	3.391	
H2B	H8A	3.148		H2B	H19A8	3.506	
H2B	H19B8	2.201		H2B	H20B8	3.408	
H2C	O2	3.053		H2C	O71	2.424	
H2C	O81	2.120		H2C	O141	3.576	
H2C	N4	3.036		H2C	N61	3.389	
H2C	C111	3.219		H2C	H4A	3.483	
H2C	H4B	2.529		H2C	H4C	2.751	
H2C	H19B8	3.146		H3A	O42	3.09(16)	
H3A	H22	3.177		H3A	H9A2	3.250	
H3A	H9B2	3.543		H3B	O42	2.98(10)	
H3B	O112	3.08(11)		H3B	O122	2.11(10)	
H3B	N72	2.62(11)		H3B	C132	3.55(12)	
H3B	C142	3.33(12)		H3B	H22	2.987	
H3B	H9A2	3.395		H3B	H132	3.278	
H4	O13	3.080		H4	C184	3.150	
H4	H18A4	3.182		H4	H18B4	2.587	
H4	H18C4	3.190		H4A	O1	2.890	
H4A	O2	2.545		H4A	O3	3.393	
H4A	N2	3.324		H4A	C1	2.228	
H4A	C2	3.045		H4A	C5	3.561	
H4A	C6	2.700		H4A	C7	2.472	
H4A	H2	3.462		H4A	H2A	2.559	
H4A	H2B	3.550		H4A	H2C	3.483	
H4B	O1	3.551		H4B	O2	3.068	


Table 14. Intermolecular contacts less than 3.60 Å involving hydrogens (continued)

atom	atom	distance		atom	atom	distance
H4B	O91	3.176		H4B	O1210	3.141	
H4B	N1	3.523		H4B	N2	2.321	
H4B	N710	3.497		H4B	C1	3.582	
H4B	C7	3.513		H4B	C20	3.048	
H4B	H1A	2.857		H4B	H2A	1.489	
H4B	H2B	2.845		H4B	H2C	2.529	
H4B	H20A	2.924		H4C	O1	2.164	
H4C	O2	2.691		H4C	O81	3.416	
H4C	O91	2.270		H4C	N2	2.959	
H4C	C1	2.823		H4C	C5	3.518	
H4C	C6	2.621		H4C	C7	3.032	
H4C	C161	3.382		H4C	H2A	2.396	
H4C	H2B	3.391		H4C	H2C	2.751	
H8A	O1312	3.104		H8A	H2B	3.148	
H8A	H9B2	3.158		H8A	H9C2	3.324	
H8B	O512	3.564		H8B	O10	3.589	
H8B	O1312	2.897		H8B	H15	3.260	
H8B	H17A	3.276		H9A	O11	3.437	
H9A	N12	3.474		H9A	N32	3.169	
H9A	H1A2	2.971		H9A	H1B2	3.316	
H9A	H3A2	3.250		H9A	H3B2	3.395	
H9A	H9B2	3.303		H9B	C82	3.326	
H9B	C92	2.577		H9B	H3A2	3.543	
H9B	H8A2	3.158		H9B	H9A2	3.303	
H9B	H9B2	1.747		H9B	H9C2	2.773	
H9C	O1312	2.863		H9C	O1412	2.789	
H9C	N812	3.192		H9C	C92	3.256	
H9C	H1A2	3.227		H9C	H1B2	3.486	
H9C	H8A2	3.324		H9C	H9B2	2.773	
H9C	H9C2	3.015		H13	N5	3.466	
H13	C197	3.295		H13	C207	3.287	
H13	H3B2	3.278		H13	H19A7	2.527	
H13	H20A7	2.456		H13	H20B7	3.541	
H15	O3	3.443		H15	O106	3.076	
H15	C106	3.502		H15	C156	3.379	
H15	C166	3.176		H15	H8B	3.260	
H15	H156	3.113		H15	H18A6	3.562	


Table 14. Intermolecular contacts less than 3.60 Å involving hydrogens (continued)

atom	atom	distance		atom	atom	distance
H17A	O512	2.684		H17A	O61	3.533	
H17A	O71	3.345		H17A	N512	3.508	
H17A	H8B	3.276		H17B	O11	3.504	
H17B	O2	3.426		H17B	O61	2.949	
H17B	O71	2.537		H17B	N61	2.827	
H17B	H22A1	3.158		H17B	H22B1	3.464	
H18A	O69	3.580		H18A	O116	2.988	
H18A	C221	3.317		H18A	H49	3.182	
H18A	H156	3.562		H18A	H22A1	2.991	
H18A	H22B1	2.808		H18B	O59	2.653	
H18B	O512	3.232		H18B	N59	3.380	
H18B	C39	3.595		H18B	C49	3.219	
H18B	H49	2.587		H18C	O69	3.569	
H18C	O61	3.077		H18C	H49	3.190	
H18C	H18C13	3.383		H18C	H21B9	3.435	
H19A	O1210	2.999		H19A	C1310	3.411	
H19A	H2B8	3.506		H19A	H1310	2.527	
H19B	O711	3.360		H19B	N28	2.801	
H19B	C198	3.027		H19B	C208	2.726	
H19B	H2A8	3.542		H19B	H2B8	2.201	
H19B	H2C8	3.146		H19B	H19B8	2.536	
H19B	H20B8	2.243		H20A	O81	2.739	
H20A	O1210	2.543		H20A	O141	3.331	
H20A	N710	3.226		H20A	C111	3.493	
H20A	C1310	2.973		H20A	H4B	2.924	
H20A	H1310	2.456		H20B	O71	3.362	
H20B	O81	3.096		H20B	O141	2.866	
H20B	N18	3.256		H20B	C198	3.019	
H20B	C208	3.492		H20B	H1B8	3.414	
H20B	H2B8	3.408		H20B	H1310	3.541	
H20B	H19B8	2.243		H20B	H20B8	3.285	
H21A	O1110	2.775		H21A	O1210	3.344	
H21A	N710	3.384		H21B	O42	2.865	
H21B	H18C4	3.435		H22A	O1	3.058	
H22A	O91	2.765		H22A	N6	3.356	
H22A	C1	3.158		H22A	C4	3.468	
H22A	C5	2.967		H22A	C6	3.032	


Table 14. Intermolecular contacts less than 3.60 Å involving hydrogens (continued)

atom	atom	distance		atom	atom	distance
H22A	C181	3.562		H22A	H17B1	3.158	
H22A	H18A1	2.991		H22B	O91	2.336	
H22B	O1110	2.280		H22B	O1210	2.998	
H22B	N710	2.699		H22B	C161	3.078	
H22B	C181	3.597		H22B	H2A	3.258	
H22B	H17B1	3.464		H22B	H18A1	2.808	


Symmetry Operators:

(1)  -X+1/2+1,-Y+1/2,-Z+1		(2)  -X+1,Y,-Z+1/2+1
(3)  -X+1/2+1,Y+1/2-1,-Z+1/2+1		(4)  X+1/2-1,Y+1/2-1,Z
(5)  X+1/2,Y+1/2-1,Z		(6)  -X+2,Y,-Z+1/2+1
(7)  X+1/2,-Y+1/2,Z+1/2		(8)  -X+1,-Y+1,-Z+1
(9)  X+1/2,Y+1/2,Z		(10)  X+1/2-1,-Y+1/2,Z+1/2-1
(11)  X+1/2-1,Y+1/2,Z		(12)  -X+1/2+1,Y+1/2,-Z+1/2+1
(13)  -X+2,-Y+1,-Z+1
